# Unexpected Malignant Diagnosis in Colonic Biopsies: Malignant Transformation of Ovarian Mature Teratomas—Two Case Reports and Review of the Literature

**DOI:** 10.1155/2015/905462

**Published:** 2015-12-31

**Authors:** Claudia P. Rojas, Parvin Ganjei-Azar, Monica T. Garcia-Buitrago

**Affiliations:** Department of Pathology, University of Miami Miller School of Medicine, Jackson Memorial Hospital, 1611 NW 12th Avenue, Holtz 2042C, Miami, FL 33136, USA

## Abstract

Colorectal adenocarcinoma is the second cause of cancer-related deaths in the United States. The occurrence of squamous cell carcinoma in the colorectum is extremely unusual. Malignant transformation from mature cystic teratoma of the ovary is a rare event. The most common transformation is squamous cell carcinoma, followed by adenocarcinoma. It occurs more often in elderly patients, who usually present with advance disease. We report two unusual cases of postmenopausal women diagnosed with squamous cell carcinoma in colon biopsies. After surgical resections, the carcinoma was proven to be the result of malignant transformation of ovarian mature cystic teratomas. Since squamous cell carcinoma of the colorectum is extremely rare, the presence of squamous cell carcinoma in a colonic biopsy in a female patient should alert the clinicians to other possible primary sites, as seen in these cases.

## 1. Introduction

Colon cancer is the third most commonly diagnosed cancer and the second leading cause of cancer death in men and women in the United States. The American Cancer Society estimated that about 136,830 people were diagnosed with colorectal cancer in the United States, and about 50,310 people were predicted to die of the disease in 2014. Adenocarcinomas are by far the most common histologic type of colorectal cancer. Squamous cell carcinoma (SCC) of the colon is a rare entity, representing only a small fraction of colorectal malignancies [1].

We report two unusual cases of postmenopausal women diagnosed with squamous cell carcinoma in colon biopsies that, in follow-up, were proven to be the result of a malignant transformation of ovarian mature cystic teratomas.

## 2. Case Presentation

### 2.1. Case  1

**Figure 1:**
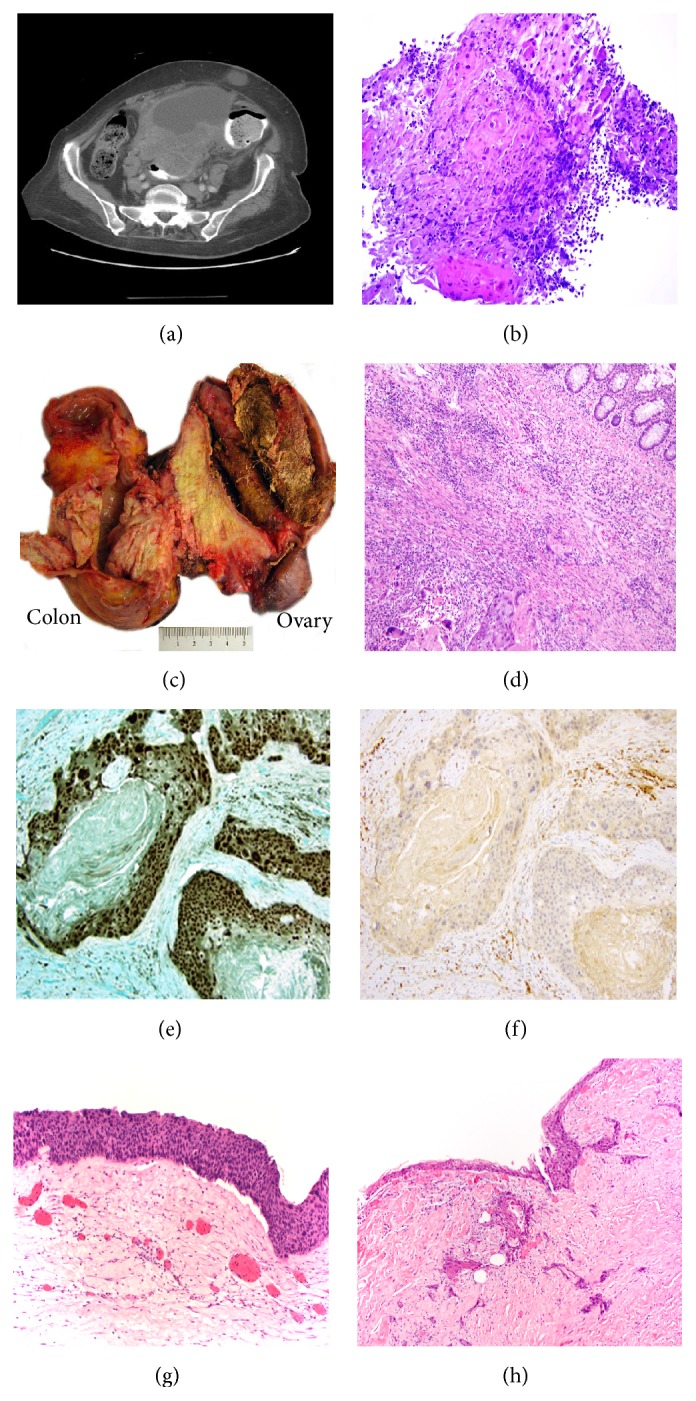
(a) Abdomen and pelvis CT showing a heterogenous mass with cystic and solid component. (b) Colon biopsy (H&E, 40x) showing a dysplastic squamous cell epithelium with keratin material, suspicious for squamous cell carcinoma. (c) Gross picture showing a cystic teratoma with sebaceous material and hair (right), the colon (left), and a firm white tan mass in between. (d) Squamous cell carcinoma (inferior left) invading into the colonic wall up to the submucosa (H&E, 20x). (e) P63 immunostain highlighting the tumor (20x). (f) Negative p16 immunostain (20x). (g) In situ squamous cell carcinoma (H&E 20x). (h) In situ squamous cell carcinoma with invasive component (H&E, 10x).

A 71-year-old Hispanic woman with history of diabetes mellitus and hypertension presented with slowly progressive constipation for 6 months, mild, dull, nonradiating, lower abdominal pain, increased abdominal girth, and 50-pound weight loss. A computed tomography scan of the abdomen and pelvis demonstrated a large 18.9 × 12.8 × 12.5 cm heterogeneous mass originating either from the right adnexa or the intestine (Figure 1(a)). The serum tumor markers were CA19.9: 119 U/mL and CEA: 7.2 ng/mL.

A colonoscopy revealed a necrotic mass located at 25 cm from the anal verge. The colonic biopsy showed minute detached fragments of dysplastic squamous epithelium, highly suspicious for squamous cell carcinoma (Figure 1(b)). She underwent a hysterectomy with bilateral salpingoophorectomy and partial colectomy.

Gross examination of the specimen revealed an 18 cm cystic mass, attached to a 14 cm segment of the colon. The cyst was filled with tan sebaceous material and black hair and was attached to the colonic wall, where a firm white 11 cm solid mass was noted (Figure 1(c)). Microscopic examination revealed an invasive moderately differentiated keratinizing squamous cell carcinoma invading up to the submucosa of the colon (Figure 1(d)). The cystic component shows a mature teratoma with in situ carcinoma in a squamous-lined cyst (Figures 1(d), 1(g), and 1(h)). The tumor cells were positive for p63 (Figure 1(e)) and negative for p16 (Figure 1(f)) by immunohistochemistry. The patient was deemed to be stage IIB and underwent 6 cycles of adjuvant chemotherapy with carboplatin and Taxotere. The tumor markers were still elevated one month after surgery; CA19-9 was 106.7 U/mL and CEA was 4.62 ng/mL. Two months later, an abdominal and pelvic CT scan revealed a new bilobed 5.5 cm mesenteric mass in the right hemipelvis, which was not separable from the adjacent bowel loops, an enlarged soft tissue mass in the left iliac fossa, and a subhepatic mesenteric mass. The patient declined any type of additional chemotherapy, moved to a different city, and was lost in follow-up.

### 2.2. Case  2

A 55-year-old Hispanic female complained of pelvic pain, loss of appetite, weakness, and 40-pound weight loss in the last 5 months. She was also noted to have a small amount of bright blood per rectum.

**Figure 2:**
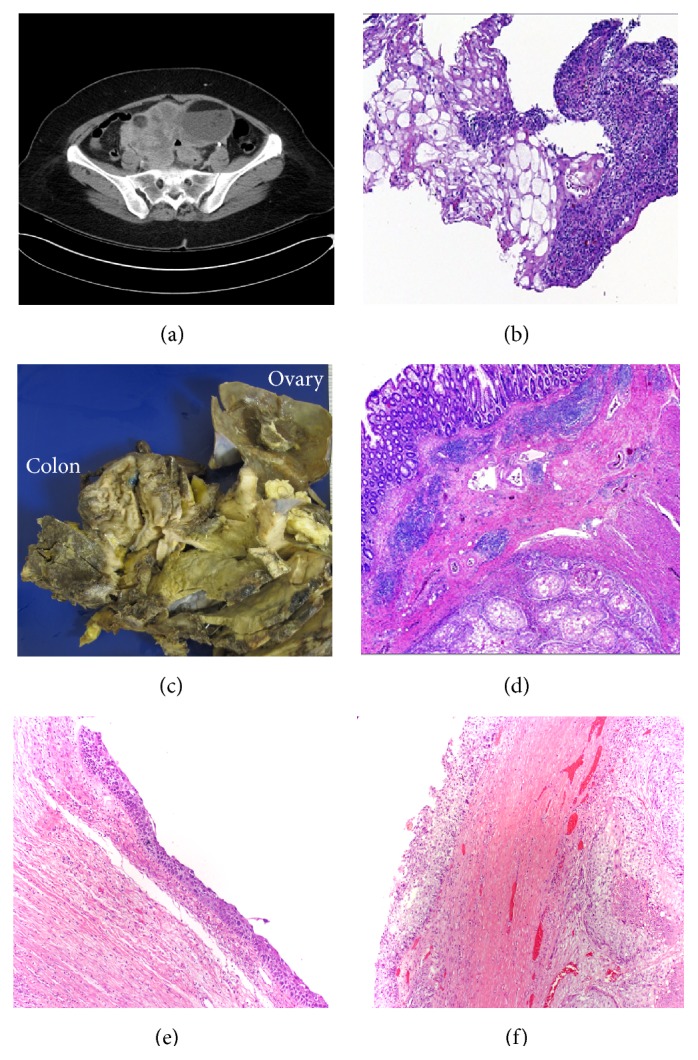
(a) Pelvis CT showing a mass with a cystic and solid component and internal septations. (b) Colon biopsy revealing an atypical squamous epithelium, suspicious for well-differentiated squamous cell carcinoma (H&E, 40x). (c) Gross picture showing a cystic ovarian mass (right) and (d) squamous cell carcinoma invading colonic wall (H&E, 20x). (e) In situ squamous cell carcinoma (H&E, 10x). (f) In situ squamous cell carcinoma and invasive component (H&E, 10x).

A computed tomography scan of the abdomen showed a 17 × 14 × 11 cm pelvic mass with cystic and solid components and internal septations, which appeared to encase the sigmoid colon (Figure 2(a)).

A colonoscopy was performed to reveal a friable and hyperemic colonic mucosa at about 20 cm from the anus. A colonic biopsy showed fragments of a highly atypical squamous epithelium, suggestive of squamous cell carcinoma. No colonic mucosa was present (Figure 2(b)). The tumor markers were CA19.9: 50.1 U/mL and CEA: 3.7 ng/mL.

The patient underwent a hysterectomy with bilateral salpingoophorectomy and rectosigmoid resection with end-to-end anastomosis.

Gross examination of the specimen revealed a 16 cm multiloculated solid and cystic adnexal mass attached to a 12 cm segment of rectosigmoid colon. The heterogeneous cystic mass was filled with adipose tissue, hair, and sebaceous material. Upon opening, the segment of colon showed a large fistulous tract that measures 2 cm in diameter (Figure 2(c)).

Microscopic examination revealed a moderately differentiated squamous cell carcinoma invading up to submucosa of the colon (Figure 2(d)) and an in situ squamous cell carcinoma component in the lining of the ovarian cyst (Figures 2(e) and 2(f)). The patient was deemed to be stage IIIB and received six cycles of chemotherapy with cisplatin and taxol. She completed the chemotherapy and, after 8 months, there was no evidence of recurrence. After that, she was lost in follow-up.

## 3. Discussion

Primary colorectal squamous cell carcinoma is an exceedingly rare malignancy representing 0.25–1 per 1000 colorectal carcinomas [2]. To date, about 120 cases of SCC have been reported in the world literature [3]. Before the diagnosis of primary SCC of colorectum is made, certain criteria must be fulfilled as established by Williams et al. in 1979 [4]. These criteria include (A) absence of squamous cell carcinoma in any other part of the body, excluding potential metastasis to the colorectal site; (B) exclusion of any proximal extension of anal squamous cell carcinoma; (C) absence of fistulous tract lined by squamous cells; and (D) confirmation of SCC by histological analysis [1, 3].

Ovarian germ cell tumors account for about 20–25% of the ovarian neoplasms. Mature cystic teratoma (MCT) is the most common ovarian germ cell tumor, representing 10–20% of all ovarian tumors. Malignant transformation (MT) occurs in less than 2% of ovarian cystic teratomas, with squamous cell carcinoma being the most common type [5]. Most MCTs are detected 15 to 20 years before they undergo a secondary malignant transformation [6]. Thus, SCC in MCT is more common in postmenopausal patients. Historically, the carcinomas are diagnosed postoperatively because there is no particular sign or symptom characteristic of a malignancy arising in mature cystic teratomas. Presenting symptoms may include abdominal pain and distension secondary to a pelvic mass. The patient with advance disease may also present with bowel or bladder symptoms.

Studies performed by Kikkawa et al. and Dos Santos et al. concluded that a tumor diameter of 10 cm or greater or a tumor demonstrating rapid growth should be a cause for concern [7, 8]. Risk factors for MT of an MCT include patient's age, tumor size, imaging characteristics, and serum tumor markers [9]. Tumor stage is the most important prognostic factor, as most of the survivors presented with early-stage disease [7]. In a review of 188 patients, Ruey-Jien et al. reported a 5-year survival rate of 75,5%; 33,3%; 20,6%, and 0% for patients with stages I, II, III, and IV, respectively [10]. The prognosis of patients with advance squamous cell carcinoma of the ovary is poor regardless of the treatment received [11]. Due to the rare incidence of malignant transformation of mature cystic teratomas, adjuvant treatment has not been standardized; however, in a review paper, Sakuma et al. recommended the use of platinum/taxane chemotherapy [12]. It has been shown that the prognosis of MT is significantly worse than that of epithelial ovarian cancer, regardless of the use of adjuvant chemotherapy or radiotherapy [8]. Ovarian carcinomas composed entirely of squamous cells arise most often in dermoid cysts but have also been reported in association with endometriosis and in a pure de novo form [13]. In order to establish the diagnosis of pure primary ovarian squamous cell carcinoma, it is necessary to exclude extragenital and genital squamous cell carcinoma as well as endometriosis [14]. These tumors are usually diagnosed after resection with extensive histologic evaluation.

Few manuscripts have reported the use of imaging modalities to diagnose MT of MCT. Kido et al. reported the MRI findings for six MCT with MT. They described solid portions present in five out of six tumors; two of them were enhanced by gadolinium to varying degrees [15]. The presence of solid, friable, or variegated components, extensive transmural extension, and direct invasion of neighboring pelvic organs suggests the possibility of MT [16–18]. In our cases, the squamous cell carcinoma infiltrated the adjacent colon, precluding the identification of the origin of the tumor at the preoperative initial biopsy.

We conclude that, in postmenopausal patients with colorectal SCC, other sources should be considered including metastasis or tumor extension of cervical or vaginal carcinoma and malignant transformation of mature cystic teratoma. In our cases, the unusual finding of squamous cell carcinoma in the colonic biopsies creates a diagnostic dilemma and the pathologist should raise the possibility of secondary involvement. Malignant transformation of a MCT must be included in the differential diagnosis when squamous cell carcinoma is found above the anal verge, especially if present in conjunction with a large pelvic mass.

## References

[1] Dyson T., Draganov P. V. (2009). Squamous cell cancer of the rectum. *World Journal of Gastroenterology*.

[2] Nahas C. S. R., Shia J., Joseph R. (2007). Squamous-cell carcinoma of the rectum: a rare but curable tumor. *Diseases of the Colon and Rectum*.

[3] Sameer A. S., Syeed N., Chowdri N. A., Parray F. Q., Siddiqi M. A. (2010). Squamous cell carcinoma of rectum presenting in a man: a case report. *Journal of Medical Case Reports*.

[4] Williams G. T., Blackshaw A. J., Morson B. C. (1979). Squamous carcinoma of the colorectum and its genesis. *The Journal of Pathology*.

[5] Rosai J. (2004). *Rosai and Ackerman's Surgical Pathology*.

[6] Russel P., Farnsworth A. (1997). Teratomas with secondary malignant transformation. *Surgical Pathology of the Ovaries*.

[7] Kikkawa F., Nawa A., Tamakoshi K. (1998). Diagnosis of squamous cell carcinoma arising from mature cystic teratoma of the ovary. *Cancer*.

[8] Dos Santos L., Mok E., Iasonos A. (2007). Squamous cell carcinoma arising in mature cystic teratoma of the ovary: a case series and review of the literature. *Gynecologic Oncology*.

[9] Chiang A.-J., La V., Peng J., Yu K.-J., Teng N. N. H. (2011). Squamous cell carcinoma arising from mature cystic teratoma of the ovary. *International Journal of Gynecological Cancer*.

[10] Chen R.-J., Chen K.-Y., Chang T.-C., Sheu B.-C., Chow S.-N., Huang S.-C. (2008). Prognosis and treatment of squamous cell carcinoma from a mature cystic teratoma of the ovary. *Journal of the Formosan Medical Association*.

[11] Gainford M., Tinker A., Carter J. (2010). Malignant transformation within ovarian dermoid cysts: an audit of treatment received and patient outcomes. an Australia New Zealand gynaecological oncology group (ANZGOG) and gynaecologic cancer intergroup (GCIG) study. *International Journal of Gynecological Cancer*.

[12] Sakuma M., Otsuki T., Yoshinaga K. (2010). Malignant transformation arising from mature cystic teratoma of the ovary: a retrospective study of 20 cases. *International Journal of Gynecological Cancer*.

[13] Ben-Baruch G., Menashe Y., Herczeg E., Menczer J. (1988). Pure primary ovarian squamous cell carcinoma. *Gynecologic Oncology*.

[14] Pins M. R., Young R. H., Daly W. J., Scully R. E. (1996). Primary squamous cell carcinoma of the ovary: report of 37 cases. *American Journal of Surgical Pathology*.

[15] Kido A., Togashi K., Konishi I. (1999). Dermoid cysts of the ovary with malignant transformation: MR appearance. *American Journal of Roentgenology*.

[16] Badmos K. B., Ibrahim O. K., Aboyeji A. P., Omotayo J. A. (2011). Squamous cell carcinoma arising in a mature cystic ovarian teratoma with bladder invasion: a case report. *African Health Sciences*.

[17] Song W., Conner M. (2012). Squamous cell carcinoma arising within a mature cystic teratoma with invasion into the adjacent small intestine: a case report. *International Journal of Gynecological Pathology*.

[18] Lee Y.-C., Abulafia O., Montalto N., Holcomb K., Matthews R., Golub R. W. (1999). Malignant transformation of an ovarian mature cystic teratoma presenting as a rectal mass. *Gynecologic Oncology*.

